# Determination of the elemental composition and antioxidant properties of dates (*Phoenix dactyliferia*) originated from different regions

**DOI:** 10.1007/s13197-020-04314-8

**Published:** 2020-03-05

**Authors:** Marzena Joanna Kuras, Monika Zielińska-Pisklak, Justyna Duszyńska, Joanna Jabłońska

**Affiliations:** Department of Biomaterials Chemistry, Chair of Analytical and Biomaterials Chemistry, Faculty of Pharmacy, Medical University of Warsaw, 1 Banacha St., 02-097 Warsaw, Poland

**Keywords:** *Phoenix dactylifera* L., Dates, Elemental composition, Antioxidant properties, Discriminant analysis

## Abstract

Due to the growing interest in running a healthy life, including the diet a special interest has been put in searching for products that are rich in nutrients, macro and micronutrients and vitamins. Dates are the fruits that meet these requirements and show multidirectional pro-health effects. These fruits are a source of potassium and other macro- and micronutrients. They have antioxidant properties thanks to the content of flavonoids and polyphenols. The elemental composition (Al, Ca, Cu, Fe, K, Mg, Mn, P, Sr and Zn) and antioxidant properties (total equivalent antioxidant capacity, total polyphenol content, total flavonoid content) of various dates from different regions of the world was determined. The results have shown that the peel and flesh of dates differ significantly in chemical composition. The peel is significantly richer in chemical components of biological importance. Discriminant analysis of the results obtained for dates originated from various regions indicated that the main factor determining the tested chemical composition is the place of cultivation, not the variety.

**Electronic supplementary material:**

The online version of this article (10.1007/s13197-020-04314-8) contains supplementary material, which is available to authorized users.

## Introduction

Dates—the fruits of *Phoenix dactylifera* L. are grown mainly in Iraq, Arabia and north Africa west to Morocco. The cultivation of the date has increased significantly in recent years. Over the past 20 years, production has almost doubled (in 1996, about 5 millon tons of dates were collected while in 2016—about 8.5 millon tons) (FAOSTAT 2019). Due to the increased production and exports, dates are now available all over the world and can be an inseparable part of the daily diet (Al-Yahyai and Manickavasagan 2012; Baliga et al. 2011).

Due to the growing interest in running a healthy life, including the diet a special interest has been put in searching for products that are rich in nutrients, macro and micronutrients and vitamins. Dates are the fruits that meet these requirements and show multidirectional pro-health effects.

The main ingredient of dates are carbohydrates (44–88%) which content depends on the stage of ripening and variety of the dates (Al-Shahib and Marshall 2003). They do not digest, but are used immediately by the body. Therefore, the dates are an ideal source of energy before training for athletes.

Additionally, dates are rich in protein (2.3–5.6%). The content of protein in dates is higher than in other fruits, eg in apples, bananas and oranges, where the protein content does not exceed 1%. There are some amino acids, for example, aspartic and glutamic acid, threonine, serine, proline, glycine and alanine are present almost exclusively in dates (Al-Shahib and Marshall 2003; Sulieman et al. 2012; Al-Showiman 1998).

The high nutritional value of dates is also associated with high fiber content. In addition to accelerating intestinal motility and thus preventing constipation, it also affects the reduction of total and LDL cholesterol levels in the blood, and additionally reduces the level of sugar (Kritchevsky 1988). Al-Shahib and Marshall (2002) examined 13 varieties of dates from different countries and showed that the fiber content varies from 6.4 to 11.5% depending on the variety and degree of maturity (Al-Shahib and Marshall 2002).

Pectin, present in dates (0.5–3.9%), also reduces risk factors associated with heart disease and diabetes, helping to reduce the amount of choleresterol in the blood (Anonymous 1987). Addictionally, dates lower the level of triglycerides. Thanks to the fact that they improve the lipid profile, they prevent atherosclerosis (Rock et al. 2009; Fayadh and Al-Showiman 1990). Studies have shown that consumption of dates by healthy people leads to decrease of serum triacyloglicerol level and serum basal oxidative stress without worsening serum glucose or lipoprotein levels (Rock et al. 2009).

Many years of research on the properties of dates have shown that they have a good antibacterial effect against *Bacillus subtilis*, *Escherichia coli*, *Shigella flexeneri*, *Pseudomonas aeruginosa*, *Staphylococcus aureus* and *Streptococcus pyogenes* (Perveen et al. 2012). Flavonoids present in dates seem to be responsible for their antifungal activity against *Candida albicans* and *Candida krusei* (Orhan et al. 2010).

Dates are a good source of minerals. Researches indicate that the concentration of minerals in the fruit of dates is affected by soil fertility, variety and ripening stage (Marzouk and Kassem 2011). The content of potassium is very high as well as calcium, magnesium and phosphorus. High potassium content and low sodium content are desirable in people suffering from hypertension. The dates flesh is also rich in iron, cobalt, copper, manganese, sodium, boron, zinc, sulfur, fluorine and selenium. Consumption of 100 g of dates provides 15% of the daily demand for selenium, copper, potassium and magnesium (Al-Showiman 1998). Many researchers focused in their experiments on determining the elemental composition of dates from various regions: Spain (Abdrabo et al. 2015), Oman (Al Farsi and Lee 2008), Saudi Arabia (Aldjain et al. 2011; Assirey 2015; Hamad et al. 2015; Mohamed 2000), Sudan (Mohamed et al. 2014; Sulieman et al. 2012). The scientists focused on determination elements that are essential for human organism as well as toxic. The results acquired by Mohamed (2000) show that the date samples of the same variety cultivated in two districts of Saudi Arabia differ by elements content, which may be due to variations in texture, structure, chemical and mineral composition of soil where the date palm was cultivated. Hamad et al. (2015) pointed out that dates from Saudi Arabia are rich in potassium (180.7–796.7 mg/100 g) followed by phosphorus (30.4–110.1 mg/100 g dry mass), magnesium (21.1–97.3 mg/100 g dry mass), and sodium (4.39–9.37 mg/100 g dry mass). They also confirmed that the content of elements varies depending on the place of cultivation. It is worth noticing that there is only one work available in which aluminum in dates has been determined (Taha and Ghtani 2015). Dates can be an excellent source of minerals in the human daily diet.

Another valuable feature of dates are their antioxidant properties which have been the subject of many studies. The most important parameter characterizing anti-oxidant properties is antioxidant activity expressed as trolox equivalent antioxidant capacity (TEAC). For a more in-depth characterization of the antioxidant properties, the total content of antioxidant compounds is determined: total polyphenols content (TPC) and total flavonoid content (TFC). Biglari et al. (2008) studied the antioxidant properties and TPC of dates from Iran. They tested soft, semi-dry and dry fruit. Their results indicate that dry dates (Kharak) have the highest antioxidant properties, TPC and TFC: 500 µmol Trolox equivalents/100 g dry weight, 141.4 mg gallic acid equivalents (GAE)/100 g dry weight and TFC 81.8 mg catechin equivalents (CEQ)/100 g dry weight, respectively. Saleh et al. (2011) examined three varieties of dates from Saudi Arabia (Ajwa, Sukari and Khalas). They compared the antioxidant properties and TPC of aqueous and alcoholic extracts. Their results show that alcoholic date extracts have lower TPC than water extracts. The Ajwa variety stood out with the highest TPC content (455.88 mg/100 g). It was also shown that the water extract of this variety has the highest antioxidant properties, which is directly correlated with TPC. Other interesting studies were conducted by Al-Turki et al. (2010). They focused on comparing antioxidant activity and TPC of dates from the USA and Saudi Arabia collected in different years of cultivation. Their determined the TPC which was in the following range: 225.0–507.0 mg GAE /100 g fresh weight. The results showed that regardless of the harvest time, dates from Saudi Arabia showed greater antioxidant properties resulting from the high TPC content.

The analysis of date fruits show that the content of antioxidant compounds depends on the variety and place of cultivation. Mansouri et al. (2005) discovered that the main phenolic acids in Algerian dates were p-coumaric, ferulic and sinapic acids, some cinnamic acid derivatives and three different iso-5-o-caffeoyl shikimic acid isomers. The phenolic acids found in Oman's fruit were ferulic acid, caffeic acid, p-coumaric acid and o-coumaric acid (Al Farsi and Lee 2008; Al Farsi et al. 2005). The main falvonoids in dates are the flavonoid glycosides of luteolin, quercetin, and apigenin (Hong et al. 2006).

A lot of research is available on the elemental composition and the antioxidant properties of dates from various regions of the world. Nevertheless, it is worth paying attention to the aspect of date sample preparation for analysis, which is a key stage affecting the obtained results. In addition, it is interesting whether the elemental composition in combination with antioxidant properties is characteristic for the date-growing region.

Our research was aimed at determining the elemental composition of dates (Al, Ca, Cu, Fe, K, Mg, Mn, P, Sr and Zn) and parameters such as: TEAC, TPC and TFC. The first stage of our research was to refine the method of preparing samples for analysis. The homogeneity of the chemical composition of dates fruits was examined. In addition, differences in the elemental composition of fruits from the same batch were determined. After analysis of the sample preparation factors affecting the analytical results, the content of elements in dates from Saudi Arabia was determined to check whether the place of cultivation of the same variety affects the elemental composition of dates. Another goal of our research was the analysis of dates available on the Polish market, originating from various regions of the world. All results of the chemical composition of dates have been subjected to discriminant analysis.

## Materials and methods

### Sample collection

Fresh date palm fruits of four different varieties (the local Arabic names are: Khalas, Sukkary, Nabtat Ali, Reshodiah) from Saudi Arabia were provided by the local manufacturer (Shimasyiah, Quassim). The fruits (2015 harvest) were used at full ripeness. There were three independent samples of Sukkary dates varied by the region of plantation (Quassim, Agil, Saada). About 30 date palm fruits of each sample were collected.

Ten samples of dates available on the Polish market were also tested. These included: from Egypt (Alrawy producer—Barhi variety), Iran (Złota Palma—Mozafati variety, Bakalland—Shahbi variety, Kimia variety, Israel (Medjool, Medjool Supreme, Medjool Blue—Medjool variety), and Tunisia (SDoukos1, SDoukos2, Bioplanet—Khouat Allig variety). Upon analysis samples were stored at − 20 °C.

### Chemicals

Deionized water used for the experiments was obtained from the Millipore system (Merck Millipore, Darmstadt, Germany). The 65% nitric acid (V) was used (J.T. Baker). The Folin-Ciocalteu reagent, sodium carbonate anhydrous, aluminum chloride anhydrous, sodium nitrate, sodium hydroxide (1 mol/l), gallic acid monohydrate were of analytical grade and purchased in POCH (Gliwice, Poland). The multielement standards, (+)-Catechin hydrate and 2,2-Diphenyl-1-picrylhydrazyl were purchased in Aldrich (Germany).

### Sample preparation

The first step of sample preparation was lyophilisation (ALPHA 1–2 LO plus (CHRIST)). The dates were vacuum dried for 48 h.

#### Sample preparation for elemental composition

The flesh and skin of the dates were separated prior to the microwave assisted digestion process (Multiwave 3000, Anton Paar, USA). The mass of 300.0 mg of the flesh was weighed in a Teflon vessel. Prior to digestion, 6 ml of the concentrated nitric acid was added to the sample. The digestion parameters are shown in Table 1S (supporting material).

After completion of the digestion process the solutions were quantitatively transferred into the measuring flasks (10 cm^3^), filled up with deionized water to the mark and mixed.

#### Extraction of antioxidants

For the determination of flavonoids, total polyphenols and antioxidant capacity, dates have been subjected to extraction.

The mass of 1.000 g of the sample was placed in a vessel and 10 ml of the methanol:water mixture (4v:1v) was added. The extraction was carried out for 72 h while shaking the samples in a laboratory shaker. Then the mixtures were filtered and the filtrate was evaporated in an oven at 40 °C for 24 h.

### Sample analysis

#### Determination of copper by graphite furnace atomic absorption spectrometry (GF AAS)

The content of copper in the date's samples was evaluated by GF AAS method (Avanta Ultra Z, GBC, Australia). The operation parametrs of the spectrometer and the temperature program are presented in tables 2S and 2S, respectively (supporting material). All the measurements were performed in triplicate.

#### Determination of aluminium, calcium, iron, magnesium, manganese, phosphorus, strontium and zinc by inductively coupled plasma atomic emission spectrometry (ICP OES)

Inductively Coupled Plasma Optical Emission Spectrometry (Optima 3100 XL, Perkin Elmer, USA) was used for the determination of the following elements: Al, Ca, Fe, Mg, Mn, P, Sr and Zn. The operating parameters of the ICP OES spectrometer are given in table 4S (supporting material). All the measurements were performed in triplicate.

#### Determination of potassium by flame atomic emission spectrometry (F-AES)

Flame photometer (BWB Technologies XP, England) was chosen for the potassium measurements. The emission was evaluated at the wavelength of 766 nm. All the measurements were performed in triplicate.

#### Determination of trolox equivalent antioxidant capacity (TEAC) with DPPH by UV–Vis spectrometry

The mass of 200.0 mg of the date's skin or flesh extract was dissolved in 10 ml of methanol.

Alcoholic solution of DPPH was prepared (concentration of 0.1351 mg/ml) by dissolving the appropriate mass of the free radical in methanol. The solution was allowed to stabilize for 3 h. Then, to the Eppendorf tubes 1 ml of the analyzed sample and 1 ml of reconstituted solution of DPPH were added. Every sample was prepared in triplicate. After exactly 30 min the absorbance at a wavelength of 517 nm was read off. The DPPH radical scavenging activity of each analytical sample was expressed as the Trolox equivalent antioxidant capacity (TEAC) (Brand-Williams et al. 1995).

#### Determination of total flavonoids content (TFC) by UV–Vis spectrometry

The mass of 100 mg of the extract was wegihed in a test tube and 6 ml of water was added in order to dissolve the sample (Chang et al. 2002).

The volume of 1.5 ml of the sample was poured to the Eppendorf tube. Then 60 μl of a 10% AlCl_3_ solution and 60 μl of a 5% NaNO_2_ solution was added to the solution. The contents of the tube were mixed and left for 5 min at room temperature. After this time, 0.4 ml of a 1 M NaOH solution was added and mixed thoroughly. The obtained solution was transferred to a cuvette and subjected to UV–Vis measurements at 510 nm. All the measurements were performed in triplicate.

The total flavonoid content was calculated from a calibration curve, and the result was expressed as mg catechin equivalent per 100 g dry weight. The experiments were performed in triplicate.

#### Determination of total phenols content (TPC) by UV–Vis spectrometry

100.0 mg of the extract was weighed and 6 ml of water was added in order to dissolve the sample. The level of total phenols in the the extracts was determined by using Folin–Ciocalteu reagent and external calibration with gallic acid (GAE) (Małyszko and Karbarz 2009).

A volume of 0.1 ml of the extract solution was withdrawn into the Eppendorf tube. Then 1.5 ml of deionized water, 0.1 ml of Folin–Ciocalteu reagent were added and the solution was mixed for 2 min. The volume of 0.3 ml of a 20% Na_2_CO_3_ solution was added to the tube and mixed thoroughly. Then the tube was placed for 20 min in the thermostat at the temperature of 40 °C. After this time, the absorbance was measured at a wavelength of 765 nm. The experiments were performed in triplicate.

### Statistical analysis

Discriminant analysis was performed by using Statistica® (v.13, Tibco®).

## Results and discussion

### The influence of the dates' homogeneity on the results obtained for elemental composition and antioxidant properties

The sample preparation process for elemental analysis is the crucial step influencing the uncertainty of the final results. Analysis of the elemental composition of fruits are particularly difficult due to the lack of sample homogeneity. This may cause difficulties in assessing and comparing the analytical results obtained by different researchers. Unfortunately, there is no uniform protocol to deal with fruit of the date palm before it is digested. What is more some authors do not give the whole procedure of date fruit sample preparation for elemental analysis (Assirey 2015). Other researches show using high speed blender in the process of sample preparation without further details (Sahari et al. 2001; Sulieman et al. 2012). The use of tools made of metal during the preparation of a sample, (eg a blender) can lead to its contamination and consequently affect the final results of determinations of selected metals. Another problem is the inhomogeneity of the fruit. The authors usually analyze the pulp independently of the stones (Abdrabo et al. 2015; Assirey 2015; Sahari et al. 2001; Sulieman et al. 2012). It is worth paying attention to the fact that the pulp analyzed is not only the soft part of the fruit but also the skin. Our research aimed to determine whether the content of elements in the flesh and peel is significantly different and if the flesh of the dates is homogenous in terms of elemental composition and antioxidant properties.

#### Elemental composition and antioxidant properties of dates' peels and flesh

**Fig. 1:**
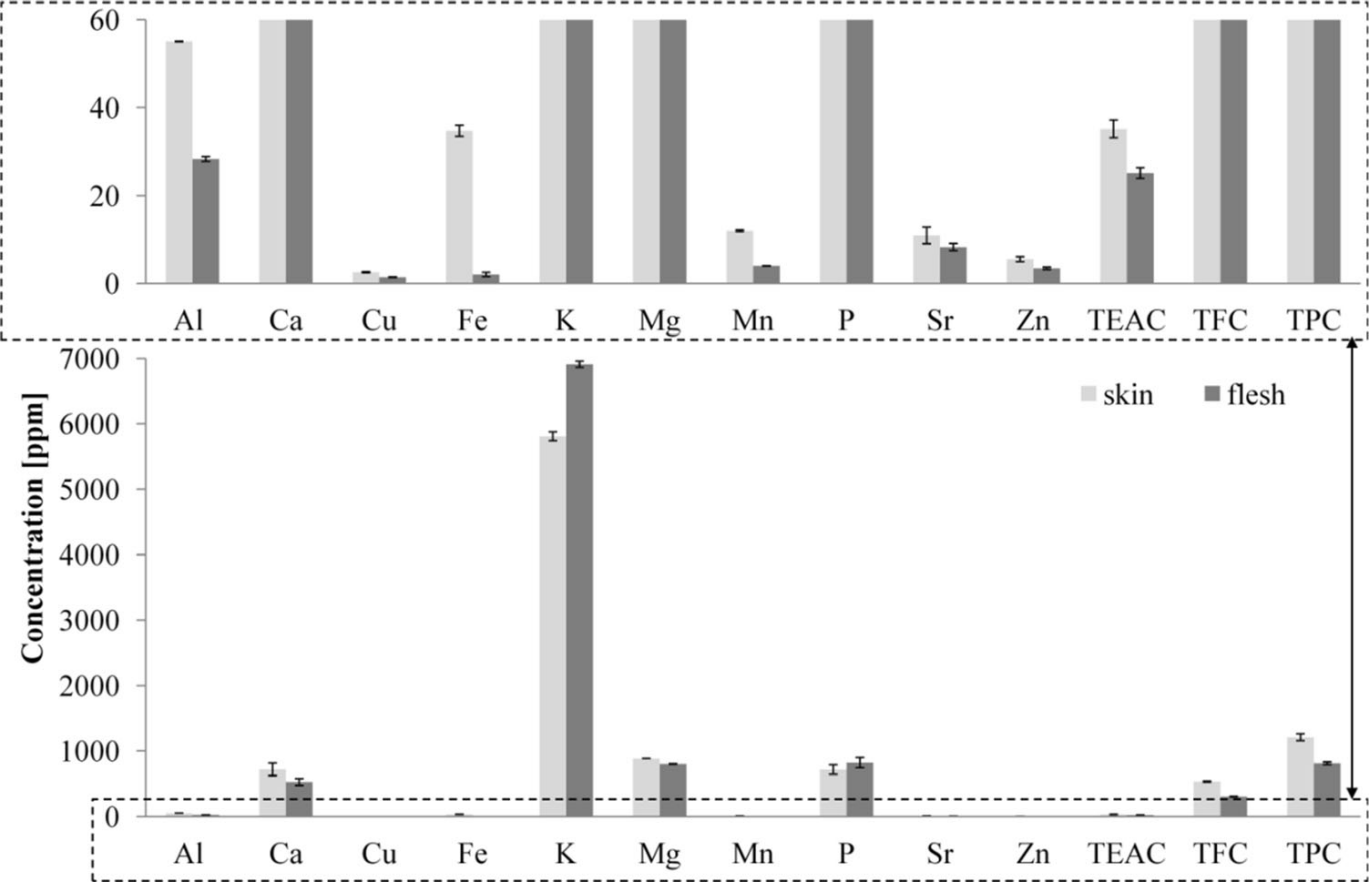
Comparison of the elemental composition, TEAC, TPC and TFC in the skin and flesh of Sukkary date from Saudi Arabia

The date fruit is composed of skin, flesh and stone. The chemical composition of those parts of the fruits may vary. There are no scientific reports available on comparing the elemental composition, total flavonoid content, phenolic content and antioxidant capacity of the skin and the date flesh. We decided to check if the values of those parameters for the flesh and corresponding peel of one date fruit differ significantly. We analyzed six independent date fruits fleshes and the peels. The samples were of different origin and variety. In Fig. 1 we present the results obtained for the date fruit from Saudi Arabia (Sukkary variety).

As shown in Fig. 1, the peel of the date is richer in the majority of elements under investigation (Al, Ca, Fe, Mg, Mn, Sr, Zn) than the corresponding flesh. These differences are statistically significant. It is worth noticing that the content of iron in the peel is at least 15 times higher than in the flesh. Similarly, there are 1.5–4.8 times more aluminum and manganese in the peel than in the flesh. Strontium is an element that in the peel is 20% more than in the flesh. These observations indicate that the fruit of the date is heterogeneous in terms of the content of elements.

Considering the antioxidant properties of the six analyzed date fruits, it can also be seen that the TEAC value is from 15 to 47% higher for the peel of the date than the flesh. In the light of these results, it is not surprising that the total content of flavonoids and phenolic acids—substances with antioxidant properties—is also significantly higher (supporting material-table 5S). The TFC values are from 307 to 542 mg and from 442 to 969 mg CEQ/100 g for the flesh and peel, respectively. The total phenolic content is 812–1498 and 998–1806 mg GAE/100 g for the flesh and peel, respectively.

Our research has shown that the fruit of the date is heterogeneous in terms of antioxidant properties and elemental composition.

This fact is crucial in the process of sample preparation for analysis. Due to the fact that the ratio of peel to pulp mass can differ between fruits and variety of dates, it is necessary to strictly define what part of the fruit had been subjected to analysis. Analyzing the mixture of the skin and the flesh can lead to deterioration of the repeatability of the analysis due to the heterogeneity of the sample.

The lack of homogeneity of the date fruit is a serious drawback in the sample preparation process. However, the knowledge that date skin is rich in elements necessary for the human body opens up wide possibilities of their application in cosmetology and pharmacy.

#### Homogeneity of the elements content in the flesh of one date fruit

The next issue worth considering is the homogeneity of the elements in the flesh of one date fruit. It is extremely important in terms of sample preparation process.

The homogeneity test for the content of elements in the flesh of the dates was carried out in order to check whether the test material is homogeneous in terms of the content of the element within one fruit and whether performing analysis on any of the dates flesh pulp results in results that reliably reflect the content of elements in the whole fruit. For this purpose, six samples of the flesh (250.0 mg) were taken from one fruit of the Sukkary variety (origin Saada) after lyophilization and were subjected to microwave digestion and analysis. The mean concentration of the elements with standard deviation and percentage coefficient of variation is shown in Table 6S (supporting material).

The results indicate that the flesh of one date is homogenous in terms of the content of most elements under investigation (Ca, Cu, Mg, K, P, Zn). The highest value of the percentage coefficient of variation was obtained for iron, strontium (16%), aluminum (14%), and manganese (11%). These values indicate that the determination of these elements in dates can be subjected to high measurement uncertainty.

Based on these results, it should be concluded that in the determination of some elements, e.g. iron, manganese, aluminum and strontium, the dispersion of results was significant. This indicates that, when analyzing those elements, the number of analyzed samples from one fruit should be increased. Taking into account the fact that in most element determinations the measurements are multi-elemental, the number of samples should be increased during routine determinations. Therefore, for the analysis, one should take many samples of the flesh from one fruit after separating the peel. What's more, the analysis requires a lot of fruit samples from one population of samples, so that the results are as representative as possible for this population of samples. Only uniform procedure yields results that can be representative and comparable to those published by different authors. What's more, in some publications we encounter methodologically questionable aspects of sample preparation process. A very important step is the washing of samples after collection. The use of tap water (Al Hooti et al. 1997) or double-distilled water (Sulieman et al. 2012; Aldjain et al. 2011; Taha and Ghtani 2015) instead of deionized water results in a relatively high level of the blank sample, which may make it impossible to detect trace elements. The drying stage is also very important. The drying conditions should be chosen so that they do not affect the original chemical composition of the samples. Air or sun drying (Taha and Ghtani 2015; Al Hooti et al. 1997) can lead to contamination of the sample. Therefore, the best way of drying seems to be lyophilization of the samples. The choice of digestion process prior to measuring the elemental composition is also important. If a closed digestion system is not used, there is a risk of loss of volatile analytes (Aldjain et al. 2011; Sulieman et al. 2012). Currently, differences in the preparation of date samples for analysis make it difficult to compare the results obtained by different researchers.

During further analyzes, measurements were taken for six samples taken from one fruit. To account for potential differences in the content of elements in different fruits belonging to the same variety—the results of the analysis include measurements for three independent fruits. The reported concentrations are the average of the results.

### Elemental composition and antioxidant properties of dates from Saudi Arabia and dates available in the Polish market

Researchers are engaged in the analysis of dates in terms of the content of elements. This is a subject that is extremely important in the light of food safety and health-promoting properties. Available publications provide information on the content of elements in dates that are necessary for the proper functioning of the human body: Ca, P, K, Na, Mg, Fe, Mn, Sr, Zn, Co, Se (Assirey 2015; Mohamed et al. 2014; Sahari et al. 2001; Sulieman et al. 2012) and toxic (As, Ba, Cd, Ni, Pb, V) (Abdrabo et al. 2015; Aldjain et al. 2011).

In order to determine the nutritional properties of the dates it is necessary to evaluate how the region of origin, climatic and growing conditions influence the content of the substances under investigation. We subjected to analysis dates belonging to the same variety (Sukkary) but from different regions of Saudi Arabia and dates from different varieties (Sukkary, Nabatat Ali, Reshodiah, Kholas) harvested in one region of Saudi Arabia.

In Table 1 we presented the element`s content and TEAC, TFC and TPC of dates from Saudi Arabia.

**Table 1:** Comparison of the elemental composition of flesh of the dates from Saudi Arabia

	Saada Sukary	Agil Sukary	Quassim Sukary	Kholas	Reshodiah	Nabatat Ali
Al	41 ± 6	25 ± 6	28 ± 0.5	28 ± 8	24 ± 0.6	38 ± 7
Ca	438 ± 2	118 ± 50	529 ± 51	392 ± 39	257 ± 12	451 ± 24
Cu	2.2 ± 0.2	2.2 ± 0.02	1.5 ± 0.07	2.9 ± 0.04	5.6 ± 0.1	1.5 ± 0.2
Fe	5.60 ± 0.9	1.1 ± 0.3	2.1 ± 0.5	4.0 ± 0.2	ND	5.5 ± 0.2
K	7950 ± 25	7843 ± 199	6914 ± 47	7015 ± 8	7390 ± 2	6060 ± 80
Mg	697 ± 19	659 ± 7	801 ± 5	788 ± 6	502 ± 2	504 ± 84
Mn	4.8 ± 0.6	7.1 ± 0.1	4.0 ± 0.04	5.8 ± 0.01	9.7 ± 0.1	3.37 ± 1.3
P	606 ± 42	794 ± 149	822 ± 79	567 ± 13	1041 ± 90	468 ± 28
Sr	3.2 ± 1.2	6.0 ± 0.3	8.3 ± 0.8	11.9 ± 1.4	4.7 ± 0.4	1.0 ± 0.3
Zn	5.5 ± 0.02	4.5 ± 0.2	3.5 ± 0.3	3.5 ± 0.3	5.8 ± 0.4	2.8 ± 0.6
TEAC	24 ± 2	38 ± 3	25 ± 1	21 ± 2	21 ± 2	17 ± 1
TFC	504 ± 8	521 ± 7	307 ± 4	384 ± 8	481 ± 19	542 ± 8
TPC	920 ± 15	1117 ± 9	812 ± 21	1253 ± 9	1498 ± 9	1056 ± 16

The concentration values expressed as mg/kg

TEAC mg trolox/100 g; values are expressed as mean ± standard error (n = 3)

The first aspect worth considering is the coefficient of variance obtained for determinations of individual elements. The results indicate large coefficients of variation for aluminum (2–24%), iron (5–27%), phosphorus (2.3–19%) and strontium (8.5–31%). For other elements, CV values usually did not exceed 10%.

Results shown in Table 2 indicate that the content of Cu, K, Mg, P, Zn, TEAC and TPC are not influenced by the harvesting and climatic conditions. The content of the other tested substances varies depending on the place of cultivation. The largest differences in content were noted for Ca, Fe and Mn. It can be due to the different composition of rain water and the soil.

**Table 2:** Comparison of the elemental composition of flesh of the Iranian and Israeli dates available on the Polish market

	Iran—Złota Palma	Iran—Bakalland	Iran—Kimia	Izrael—Medjool Supreme	Izrael—Medjool	Izrael—Medjool Blue
Al	34 ± 1	29 ± 3	27 ± 2	25 ± 1	26 ± 1	23 ± 0.2
Ca	155 ± 8	603 ± 21	533 ± 19	207 ± 14	338 ± 173	626 ± 172
Cu	4.3 ± 0 .021	3.3 ± 0.025	6.3 ± 0.065	2.6 ± 0.027	1.7 ± 0.038	7.0 ± 0.093
Fe	2.9 ± 1.3	6.7 ± 1.6	8.7 ± 2.9	ND	0.9 ± 0.2	0.8 ± 0.1
K	8521 ± 105	7431 ± 10	8280 ± 426	7435 ± 140	9303 ± 64.0	7276 ± 343
Mg	479 ± 3	456 ± 2	610 ± 70	717 ± 10	760 ± 55	892 ± 116
Mn	9.0 ± 0.09	5.3 ± 0.03	12 ± 0.11	4.5 ± 0.06	3.7 ± 0.09	5.1 ± 0.01
P	431 ± 32	474 ± 81	795 ± 58	595 ± 44	678 ± 59	581 ± 113
Sr	4.4 ± 1.1	7.4 ± 3.5	12 ± 1.8	8.3 ± 0.7	8.3 ± 2.0	11 ± 3.1
Zn	3.6 ± 0.5	4.4 ± 2.4	11 ± 1.0	3.2 ± 0.4	3.0 ± 1.2	3.8 ± 1.3
TEAC	36 ± 3	44 ± 4	73 ± 6	33 ± 3	56 ± 5	54 ± 4
TFC	317 ± 11	404 ± 8	678 ± 6	313 ± 9	452 ± 5	426 ± 5
TPC	861 ± 12	942 ± 18	1565 ± 24	978 ± 21	1300 ± 11	905 ± 10

The concentration values expressed as mg/kg

TEAC mg trolox/100 g; values are expressed as mean ± standard error (n = 3)

It is worth noticing that the Reshodiah variety is the richest in copper, phosphorus, zinc and TPC among the other tested varieties. The highest content of potassium and TEAC was recorded for Sukkary variety. A good source of magnesium can be dates of Sukkary and Kholas variety.

It is also interesting what nutritional properties have dates from different regions of the world available on the Polish market. To explore this topic, we decided to analyze the samples of dates available in Polish stores. The analytical results are presented in Tables 2 and 3.

**Table 3:** Comparison of the elemental composition of flesh of the Tunisian and Egyptian dates available on the Polish market

	Tunezja—SDoukoss1	Tunezja—SDoukoss2	Tunezja—Bioplanet	Egypt
Al	24 ± 2	25 ± 0.2	27 ± 2	31 ± 3
Ca	239 ± 24	269 ± 30	334 ± 33	399 ± 41
Cu	2.9 ± 0.03	1.2 ± 0.02	2.9 ± 0.2	1.6 ± 0.1
Fe	3.5 ± 0.2	2.3 ± 0.4	ND	5.4 ± 2.6
K	9110 ± 492	10,550 ± 1121	7633 ± 690	7752 ± 125
Mg	605 ± 41	786 ± 147	447 ± 53	683 ± 56
Mn	4.3 ± 0.07	3.7 ± 0.02	4.8 ± 0.02	4.4 ± 0.006
P	674 ± 72	616 ± 120	604 ± 112	651 ± 87
Sr	4.8 ± 2.3	4.3 ± 0.7	3.3 ± 0.5	12 ± 0.2
Zn	2.4 ± 0.6	3.6 ± 0.2	0.6 ± 0.2	3.3 ± 0.2
TEAC	75 ± 6	74 ± 6	72 ± 6	91 ± 7
TFC	1005 ± 10	839 ± 6	623 ± 8	751 ± 7
TPC	1253 ± 11	1342 ± 23	842 ± 18	1411 ± 16

The concentration values expressed as mg/kg

TEAC mg trolox/100 g; values are expressed as mean ± standard error (n = 3)

Analyzing the results in Tables 2 and 3, one can observe that in the sample of the Kimia variety from Iran, the highest content of such elements as iron, manganese, phosphorus, strontium and zinc occurs. In addition, the highest TPC value (1565 mg/kg) was determined for this sample. The lowest content of elements such as iron, magnesium, strontium and zinc was observed for a sample from Tunisia (Bioplanet). This sample is also characterized by the lowest total polyphenols content (TPC) of all the tested samples.

The results obtained for the Medjool variety from Israel are comparable with the results available in the publications for the following elements: Ca, Cu and Mn. The potassium content determined within our tests (concentration range 7276–9303 mg/kg) was significantly higher than in the available publication. A similar trend was observed for Mg, Sr and Zn. The iron content in the samples we tested was significantly lower, which may indicate the effect of the sample preparation process on the analytical results obtained (Abdrabo et al. 2015).

Sahari et al. (2001) determined selected elements in the Kholas variety. Our results for magnesium and calcium are consistent with the values obtained by his group. The only difference is in the content of potassium (in our work we obtained the content of 701.5 ± 0.8 mg/100 g, Sahari et al. (2001) determined the content of 434.97 ± 13.30 mg/100 g).

The tested dates samples present on the Polish market are characterized by a varied content of the parameters determined. Dates are considered to be an excellent source of potassium. Those present in Poland are not different in terms of the content of this element. Its average content in all tested samples was 8329 ± 1057 mg/kg, with the highest content for SDoukoss2 (Tunisia)—10,550 mg/kg and the lowest for Bakalland (Iran)—7431 mg/kg. The high content of potassium in dates helps to maintain normal blood pressure and protects against hypertension (Al-Shahib and Marshall 2002). The concentration range of the other determined elements is as follows: magnesium (447–892 mg/kg) > phosphorus (431–795 mg/kg) > calcium (155–626 mg/kg) > aluminum (23–41 mg/kg) > manganese (3.7–12 mg/kg) > strontium (3.3–12 mg/kg) > zinc (0.6–11 mg/kg) > iron (below LOD–8.7 mg/kg) > copper (1.6–7.0 mg/kg).

It is worth paying attention to the results obtained for the sample of Bioplanet dates. These dates were purchased in the organic store (assuming that cultivation was carried out in accordance with the requirements for organic farming). In terms of the content of essential elements, they are not superior to other samples. For example, the content of potassium, calcium and zinc is up to 30%, 50%, 95% lower than in other researched dates available on the Polish market, respectively. In addition, the iron content in those dates is negligible.

In available publications characterizing the elemental composition of dates, there is only one considering the content of aluminum (Taha and Ghtani 2015). The authors analyzed dates from Saudi Arabia and the content of Al was in the range from 13.6 to 74.85 ppm. Our results show that the content of Al in analyzed date samples do not exceed 41 ppm. This incompatibility of the results may be due to differences at the sample preparation stage. (lyophilization and the use of deionized water can significantly reduce aluminum contamination, which results in lower levels of this element). It should be noted that compared to vegetables, the determined aluminum content in dates is significantly lower.

Njenga et al. (2007) analyzed 17 vegetables from Kenya and determined the aluminum content. Obtained aluminum contents were significantly higher than the aluminum content in dates. The least concentration of Al was obtained for carrot (96 mg/kg of dry matter) and the highest for parsley (1062 mg/kg of dry matter). The aluminum contents in different fruit varietes from China was acquired by Liang et al. (2019). The results show that fruits have relatively low level of aluminium. The richest fruit in this element is apple (with average value of 5.3 ± 5.2 mg/kg and maximum value of 17.9 mg/kg). In fruits such as pear, watermelon and peach the maximum content of Al is 4.6 mg/kg. These results indicate that in comparison with other fruits the aluminum content in dates is significantly higher. Nevertheless, the level of aluminum in fruits and vegetables is considered low.

The antioxidant properties of dates have been the subject of experiments carried out by many researchers. In 2002, the in vitro study showed for the first time that aqueous extracts of dates had the ability to remove peroxide and hydroxyl radicals. In addition, it inhibited lipid peroxidation caused by iron ions and oxidation of proteins (Vayalil 2002). Antioxidant properties are associated with the presence of phenolic compounds in dates, such as, for example, ferulic acid, p-coumaric acid or sinapine acid, as well as flavonoids and procyanidins (Mansouri et al. 2005). Flavonoids exhibit a wide variety of health-promoting activities. They have the ability to scavenge free radicals, inhibit the activity of oxidases and metal chelation. Studies have shown that a diet rich in plant products with a high content of flavonoids, such as fruit, vegetables, wine or tea, reduces the risk of coronary heart disease. Proanthocyanidins, which are epicatechin polymers (flavanols) affect many phenomena such as: inflammatory processes in the vessels, platelet aggregation, vascular endothelial dysfunction, which is also manifested by cardioprotective effects (Tiwari and Husain 2017). Studies conducted by Vayalil (2002) have demonstrated the antimutagenic and antioxidative effects of aqueous extract from dates. These studies have led to further studies on the antioxidant properties of dates from various regions of the world. In our research the highest antioxidant properties were observed for samples from Tunisia, Iran (Kimia) and Egypt. The TFC was also the highest in these samples, which indicates a strong correlation between TFC and TEAC.

Mohamed et al. (2014) tested Sudanese dates (peel and flesh). They determined TPC: values from 35.82 ± 5.01 to 199.34 ± 9.51 mg GAE/100 g DW and TFC: values from 1.74 ± 0.04 to 3.39 ± 0.09 mg CE/100 g. They also determined antioxidant activities of the studied date varieties: FRAP, chelation of Fe (II) and scavenging of H_2_O_2_. Al-Mamary et al. (2014) tested the in vitro antioxidant activity of different types of palm dates syrups. They tested dates from Saudi Arabia, Iraq and Yemen. The TPC of the fruits from Yemen, Iraq and Saudi Arabia was 769.6 ± 7.2, 434.3 ± 1.8 and 600.3 ± 4.0 mg CE/100 g, respectively. The TFC of the fruits from Yemen, Iraq and Saudi Arabia was 554.0 ± 8.7, 310.5 ± 2.8 and 372.7 ± 1.7 mg QE/100 g, respectively (Al-Mamary et al. 2014).

The results obtained as part of this work indicate that the place of growing dates in a given country and the variety has no significant impact on the antioxidant properties of the flesh of the dates. The TEAC value did not differ significantly for all samples from Saudi Arabia and was in the range of 17–38 mg/100 g (mean 24 ± 7 mg/100 g). The obtained TFC and TPC values for dates from Saudi Arabia were 30.7–54.2 mg/kg and 81.2–149.8 mg/100 g, respectively. The results are not consistent with the results obtained by Al-Mamary et al. (2014). They are significantly lower. This may be due to differences in sample preparation. The time and medium of extraction have a significant influence on extraction efficiency. What's more, in our work, the extract of dates flesh was examined, excluding the peel, which is rich in ingredients with antioxidant properties.

Our TFC determination results of Tunisian dates are in agreement with those obtained by Chaira et al. (2009) The acquired TFC values were in the range of 6.23–10.05 mg/100 g. As for TPC, in our work we obtained slightly higher levels, which amounted to 84.2–134.2 mg/100 g.

Biglari et al. (2008) tested the antioxidant properties of the dates from Iran. The sample preparation process involved pitting, crushing and cutting into small pieces with a sharp knife and dry-blending with a blender before extraction with ml methanol–water. The reported results for TPC and TFC are in agreement with ours. For Iranian dates that we analyzed, the TPC values were 86.1–156.5 mg/100 g and TFC values were in the range of 31.7–67.8 mg/100 g of dry matter.

Dates that are available on the Polish market with the highest antioxidant properties come from Egypt (91 mg trolox/100 g). The lowest TEAC value was obtained for the sample Medjool Supreme from Israel (33 mg trolox/100 g). It should be noted that apart from samples from Egypt, dates from Tunisia showed relatively high antioxidant activity. For the Bioplanet sample, dates from the organic food store, the content of antioxidant substances is lower than in the other dates tested. The total polyphenol content and total flavonoid content is up to 46% and 40% lower. This indicates that the results of fruit analysis from various sources available on the market can help consumers choose products rich in antioxidant substances. This is extremely important because it has been shown that the phytochemicals present in date fruits have antioxidant properties that can lead to lower incidence and lower mortality due to degenerative diseases in humans (Baliga et al. 2011; Vayalil 2002). Dates, as fruits with high content of essential elements and substances with antioxidant activity, can be considered as a food additive and as functional food.

### Discriminant analysis of the results obtained for dates originated from different regions of the world

The analysis of many parameters for the tested samples poses many difficulties. It is difficult to detect significant differences between samples or groups of samples and determine unequivocally whether there are significant differences in the chemical composition of samples of dates from different regions of the world. In such cases, advanced statistical methods, for example discriminant analysis, are very helpful. It allows to determine whether on the basis of the obtained results it is possible to isolate groups of samples and whether these groups reflect the origin of dates.

Discriminant analysis has proved its worth in distinguishing the place of cabbage cultivation (China or Korea) on the basis of the analysis of the composition of volatile compounds by EN-MS (Lee et al. 2017). Discriminant analysis is also helpful in checking the authenticity of food, as well as forensic analysis to identify the cannabis cultivation region (Murphy et al. 2010; Kuras and Wachowicz 2011). In our work, discriminant analysis was used to determine whether the chemical composition of dates samples originated from various regions differs significantly.

Discriminant analysis has been performed for 15 samples of dates from five different countries.

The standardized coefficients of discriminant functions, which allow to determine which variables have the largest share in the differentiation of samples of dates (Table 7S – supporting material).

The parameter *cumulative ratio* provides information on how many percent of the variation describes certain discriminant functions. The first discriminant function explains 71% of variation, the second 18% and the others only 11%. Functions U1 and U2 contain 89% of the total discriminating power, so the third and fourth functions (U3 and U4) were excluded from further considerations since they explain a negligible percentage of variation.

The element that has the largest contribution to the discriminatory function U1 is iron, for which the highest coefficient was obtained. Subsequent factors that have a significant contribution to U1 are TFC, phosphorus, zinc and manganese. On the other hand, the biggest contribution to explaining variability with U2 are magnesium, strontium, potassium, zinc and phosphorus.

**Fig. 2:**
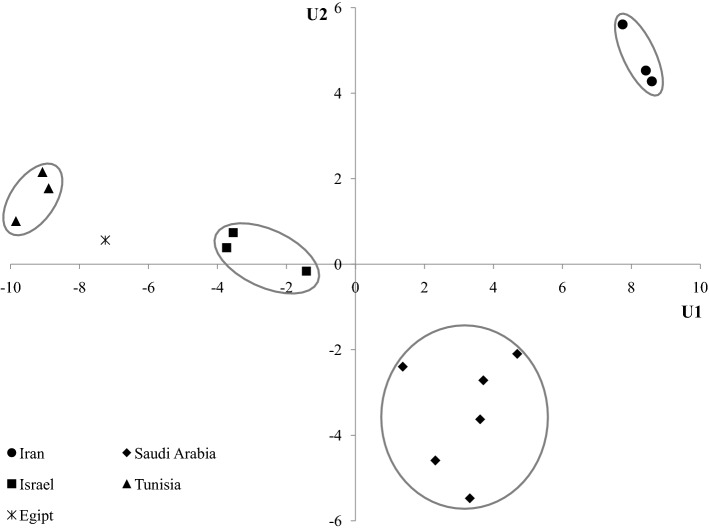
Scatter diagram of dates samples harvested in different countries

Figure 2 show a scatter diagram of discriminant functions.

Despite the limited number of samples, the discriminant analysis showed clear differences between the groups of dates of various origin. Additionally, it can be noticed that the points in a particular group are not dispersed, which indicates their similarity within the group. The results clearly indicate that the place of cultivation determines the elemental composition and antioxidant properties of dates. This confirms that the chemical composition of dates from different countries is clearly characteristic of a given harvesting region. Climatic conditions, cultivation methods, soil composition are some of the parameters that determine the chemical composition of the fruit. Identification and analysis of chemical parameters can help producers in obtaining fruit with a higher content of substances with a positive effect on our body.

Discriminant analysis of results is also invaluable in identifying the origin of dates and may be used in the future to check the authenticity of dates on the food market.

## Conclusion

Our work includes the analysis of the elemental composition and antioxidant properties of dates from Saudi Arabia and available on the Polish market. A large part of the research was focused on the process of preparing the sample for analysis. It turned out that the dates are heterogeneous in terms of elemental composition, TEAC, TFC and TPC. Significant differences in these parameters occur between the peel and the flesh. What's more, even the fruit flesh of one batch shows inhomogeneity in terms of the content of the substances tested. This indicates the need to develop a uniform protocol for dealing with samples of dates before analysis, so that the obtained results could be compared to those obtained by other researchers.

Date peels are rich in substances necessary for the human body. The content of those substances in peels is significantly higher than in the corresponding pulp. This inhomogeneity in the chemical composition of the date fruit is a disadvantage in the process of sample preparation for analysis. However the fact that the date peel is rich in elements necessary for the human body opens up wide possibilities of their application in cosmetology and pharmacy.

The content of elements in dates available on the Polish market, arranged by decreasing concentration is as follows: K > Mg > P > Ca > Al > Mn > Sr > Zn > Fe > Cu. Dates are considered an excellent source of potassium. On the Polish market, the Tunisian dates are the richest in this element (SDoukoss). It is worth adding that the dates from the organic store did not show higher content of substances of biological importance than the other dates analyzed.

The results of our research indicate the diversification of the chemical composition of dates from different regions. Discriminant analysis of results obtained for different varieties of dates from Saudi Arabia showed one isolated group of samples. This indicates that the main determinant of the chemical composition of dates is the place of cultivation, i.e. climatic conditions. Analysis of elemental composition and antioxidant properties in combination with discriminant analysis may in the future be used to test the authenticity of dates on the market.

## Electronic supplementary material

Below is the link to the electronic supplementary material.

Supplementary file1 (DOCX 24 kb)
