# Supplementary material for: Determination of the elemental composition and antioxidant properties of dates (Phoenix dactyliferia) originated from different regions
Source: J Food Sci Technol. 2020 Mar 5;57(8):2828–39. doi: 10.1007/s13197-020-04314-8 (PMC7316905; doi:10.1007/s13197-020-04314-8)
Supplement: Supplementary file 1 — Supplementary file1 (DOCX 24 kb) [file 13197_2020_4314_MOESM1_ESM.docx]

Table 1S. Microwave digestion programme.

| **Stage** | **Power [W]** | **Time [min]** |
| --- | --- | --- |
| 1 | 0 - 600 | 20 |
| 2 | 600 | 10 |
| 3 | 600 - 1200 | 10 |
| 4 | 1200 | 15 |

Table 2S. The operation parameters of the GF AAS spectrometer for Cu determination.

| **Parameter** | **Value** |
| --- | --- |
| Wavelength [nm] | 324.7 |
| Lamp current [mA] | 5 |
| EHT voltage [V] | 300 |
| Slit width [nm] | 0.5 |
| Magnetic Field Strength [T] | 1.00 |

Table 3S. Graphite Furnace temperature program implemented in copper determination by GF AAS (sample volume: 20µl).

| Stage | Temprature [°C] | Ramp time [s] | Hold time [s] |
| --- | --- | --- | --- |
| 1 | 110 | 35 | 15 |
| 2 | 130 | 5 | 5 |
| 3 | 800 | 10 | 10 |
| 4 | 800 | 0 | 1 |
| 5 | 2100 | 0.6 | 0.9 |
| 6 | 2500 | 0.2 | 0.8 |

Table 4S. The operation parameters of the ICP OES spectrometer for determination of Al, Ca, Fe, Mg, Mn, P, Sr and Zn.

| **Parameters** | **Conditions** |
| --- | --- |
| Delay time [s] | 65 |
| Plasma View | axial |
| Peristaltic pump flow rate [ml/min] | 0.65 |
| RF power [W] | 1400 |
| Plasma gas flow [L/min] | 15 |
| Nebuliser gas flow [L/min] | 0.65 |
| Replicates | 3 |
| Internal Standard | Y |
| Wavelength [nm] | Al: 396.153 Ca: 317.930  Fe: 238.204 Mg: 285.207  Mn: 257.604 P: 213.617  Sr: 232.235 Zn: 206.200  Y: 371.029 |

Table 5S. Comparison of the TEAC parameter of flesh and corresponding skins of the dates from Saudi Arabia. TEAC parameter as mass of trolox in mg per 100 g sample.

|  |  | **TEAC**  **[mg trolox/100g]** | **TFC  [mg CEQ/100g]** | **TPC  [mg GAE/100g]** |
| --- | --- | --- | --- | --- |
| **Kholas** | flesh | 20.8 ± 1.7 | 384 ± 7.8 | 1253 ± 8.8 |
| **(47%)*** | skin | 30.5 ± 1.0 | 443 ± 5.6 | 1399 ± 53 |
| **Quassim** | flesh | 25.2 ± 1.2 | 307 ± 4.1 | 812 ± 21 |
| **(40%)*** | skin | 35.3 ± 2.8 | 538 ± 7.0 | 1210 ± 52 |
| **Reshodiah** | flesh | 20.8 ± 1.6 | 481 ± 19 | 1498 ± 8.9 |
| **(36%)*** | skin | 28.3 ± 1.5 | 969 ± 9.2 | 1806 ± 39 |
| **Agil** | flesh | 38.2 ± 3.3 | 521 ± 6.8 | 1117 ± 9.2 |
| **(22%)*** | skin | 46.7 ± 2.3 | 587 ± 10 | 998 ± 22 |
| **Saada** | flesh | 23.6 ± 1.6 | 504 ± 8.3 | 920 ± 15 |
| **(43%)*** | skin | 33.6 ± 2.9 | 442 ± 6.5 | 1201 ± 43 |
| **Nabatat Ali** | flesh | 17.4 ± 1.2 | 542 ± 8.1 | 1056 ± 16 |
| **(15%)*** | skin | 19.9 ± 1.1 | 687 ± 5.3 | 1195 ± 12 |

* percent difference between TEAC in the peel and flesh.

Table 6S. Concentration of elements in the date flesh samples of one fruit [ppm].

The concentration values expressed as mg/kg. Results for Saada sample.

|  | **Saada - one fruit** | |  |
| --- | --- | --- | --- |
| **Element** | **C [ppm]** | **SD [ppm]** | **CV [%]** |
| **Al** | 40.7 | 5.7 | 14 |
| **Ca** | 438 | 2.1 | 0.5 |
| **Cu** | 1.43 | 0.15 | 2.4 |
| **Fe** | 54 | 8.8 | 16 |
| **Mg** | 697 | 19 | 2.7 |
| **K** | 7879 | 189 | 3.1 |
| **Mn** | 5.69 | 0.17 | 11 |
| **P** | 606 | 42 | 6.9 |
| **Sr** | 3.2 | 0.5 | 16 |
| **Zn** | 5.5 | 0.02 | 0.4 |

Table 7S. Standardized coefficients (b`) of discriminant functions.

| **Parameter** | **U1** | **U2** | **U3** | **U4** |
| --- | --- | --- | --- | --- |
| **TFC** | -2,70 | 0,27 | 0,15 | -0,74 |
| **TPC** | 0,91 | -1,35 | -0,94 | 0,22 |
| **TEAC** | -1,77 | 0,15 | 0,83 | 0,98 |
| **K** | 0,35 | 2,19 | -0,13 | -0,23 |
| **Mg** | 0,91 | -3,18 | 0,78 | -0,46 |
| **Fe** | 5,17 | -0,80 | 2,12 | -0,58 |
| **Cu** | 0,92 | 0,96 | -1,36 | -0,57 |
| **Mn** | 2,33 | -1,51 | 3,00 | -0,33 |
| **P** | 2,60 | -1,56 | 1,18 | -0,81 |
| **Sr** | -1,96 | 2,54 | -0,51 | 1,04 |
| **Zn** | -2,55 | 1,88 | -2,83 | 0,79 |
| **Al** | 0,00 | 0,00 | 0,00 | 0,00 |
| **Ca** | 0,00 | 0,00 | 0,00 | 0,00 |
| **Eigenvalue** | 54,61 | 13,72 | 7,23 | 1,37 |
| **Cumulative proportion** | 0,71 | 0,89 | 0,98 | 1,00 |
